# Bacterial Reaction Centers Purified with Styrene Maleic Acid Copolymer Retain Native Membrane Functional Properties and Display Enhanced Stability**

**DOI:** 10.1002/anie.201406412

**Published:** 2014-09-11

**Authors:** David J K Swainsbury, Stefan Scheidelaar, Rienk van Grondelle, J Antoinette Killian, Michael R Jones

**Affiliations:** School of Biochemistry, University of Bristol, Medical Sciences Building, University WalkBristol BS8 1TD (UK); Membrane Biochemistry & Biophysics, Utrecht University, Bijvoet Center for Biomolecular ResearchUtrecht (The Netherlands); Division of Physics and Astronomy, VU University AmsterdamDe Boelelaan 1081, Amsterdam 1081 HV (The Netherlands)

**Keywords:** detergents, membrane proteins, nanodiscs, reaction centers, styrene maleic acid

## Abstract

Integral membrane proteins often present daunting challenges for biophysical characterization, a fundamental issue being how to select a surfactant that will optimally preserve the individual structure and functional properties of a given membrane protein. Bacterial reaction centers offer a rare opportunity to compare the properties of an integral membrane protein in different artificial lipid/surfactant environments with those in the native bilayer. Here, we demonstrate that reaction centers purified using a styrene maleic acid copolymer remain associated with a complement of native lipids and do not display the modified functional properties that typically result from detergent solubilization. Direct comparisons show that reaction centers are more stable in this copolymer/lipid environment than in a detergent micelle or even in the native membrane, suggesting a promising new route to exploitation of such photovoltaic integral membrane proteins in device applications.

Biophysical characterization of integral membrane proteins and their use in biotechnology usually requires their removal from the native lipid-bilayer environment using detergents. The identification of the best detergent for purification of a given protein typically involves a process of trial and error, and the final choice may not fulfill all requirements for optimal stability or native functionality.^1^ An important concern is the extent to which the transfer of a protein to a detergent environment strips away structurally and functionally important annular lipids. A recently developed alternative is the use of an amphipathic styrene maleic acid (SMA) copolymer (Figure 1 A) that is able to remove the protein from a membrane with its associated lipids intact in the form of a protein/lipid nanodisc bound by the polymer.^2^–^5^ This approach has been used to successfully solubilize membrane proteins from artificial liposomes^2^, ^6^ and native membranes,^7^, ^8^ and is one of a number of alternatives being developed for housing integral membrane proteins outside the native membrane.^9^

**Figure 1:**
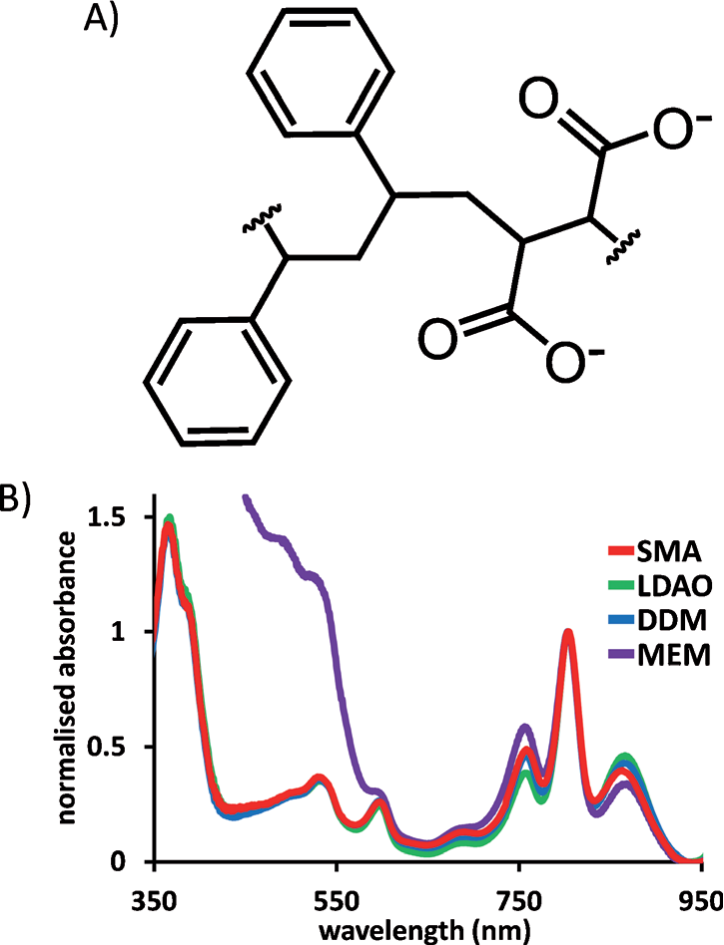
A) Chemical structure of the average repeating unit of the SMA copolymer. B) Absorbance spectra of *Rba. sphaeroides* RCs in different environments after normalization and correction for scattering (for details see Experimental Procedures in the Supporting Information).

In the present report, a SMA copolymer (Figure 1 A) was used to purify the photoreaction center (RC) from the purple bacterium *Rhodobacter* (*Rba.*) *sphaeroides*. This classic alpha-helical integral membrane pigment-protein complex, which has a strong, distinctive bacteriochlorin absorbance spectrum that provides information on both structural and functional integrity, has been structurally characterized^10^ and subjected to an extensive biophysical characterization.^11^ It conducts an extremely efficient charge separation that lends itself to applications in photovoltaics, molecular electronics, biosensing, and photocatalysis.^12^ However, such applications require the preparation of large amounts of protein that is structurally stable and optimally active. This presents challenges because the functional properties of the RC are known to be modulated when it is removed from the native membrane,^13^–^15^ and its stability is known to be dependent on the detergent/lipid environment.^16^

To determine the usefulness of the SMA copolymer as a new vehicle for housing integral membrane proteins such as RCs, the properties of SMA-purified RCs were systematically compared with those of RCs purified in the commonly used detergents *N*,*N-*dimethyldodecylamine *N*-oxide (LDAO) and *n*-dodecyl β-d-maltoside (DDM), as well as with those of RCs in intact native photosynthetic membranes. The latter was possible through the use of a strain of *Rba. sphaeroides* that lacks the genes that encode the native light-harvesting complexes, leaving the RC accessible as the sole bacteriochlorin-containing complex in the membrane.^13^ RCs modified with a His_10_ tag were solubilized by the addition of SMA, LDAO, or DDM, and purified using nickel affinity and size-exclusion chromatography (see the Supporting Information).

The strong absorbance spectrum of the RC provides a simple way to monitor its properties. In the near-infrared region, it comprises three main bands that arise from the bacteriochlorin cofactors (Figure 1 B), the relative intensities and wavelength maxima of which are known to be modulated somewhat by the detergent/lipid environment of the protein.^13^, ^14^ The SMA-solubilized RCs had an absorbance spectrum that was similar to that of RCs in native membranes or solubilized in either LDAO or DDM, showing that it is structurally intact in the SMA/lipid nanodiscs (Figure 1 B). Small differences in intensity of the bands at 865 and 760 nm in the four preparations are a consequence of the sensitivity of cofactors at, or close to, the surface of the protein to its detergent/lipid environment.

The purity of LDAO- or DDM-solubilized RCs can be conveniently quantified from the ratio between protein absorbance at 280 nm and bacteriochlorophyll absorbance at 802 nm.^17^ Pure RCs gave a ratio of approximately 1.3, as determined by SDS-PAGE.^17^ For SMA-solubilized RCs purified to the same degree, this ratio was around 1.5 (see Figure S1 in Supporting Information for discussion).

Analysis by dynamic light scattering (DLS) showed that the average diameter of purified SMA/lipid/RC nanodiscs was 12.2±7.1 nm, significantly larger than that of RCs in micelles formed from DDM (7.8±4.8 nm) or LDAO (5.1±2.7 nm). Images of SMA/lipid/RC nanodiscs obtained by negative staining transmission electron microscopy (TEM; Figure 2 A) showed particles with a diameter of approximately 12–15 nm, in good agreement with the DLS data. Pretreatment of the discs with 5 nm Ni-nitrilotriacetic acid functionalized gold nanoparticles allowed detection of the RC His_10_ tag. The off-center position of this gold nanoparticle in most labeled nanodiscs (Figure 2 A) is consistent with the expected off-center location of the His_10_ tag on the periplasmic surface of the RC. This point is illustrated in the schematic model of a gold-labeled SMA/lipid/RC nanodisc in Figure 2 B. It may also indicate that individual RCs do not necessarily reside at the center of their SMA/lipid nanodisc.

**Figure 2:**
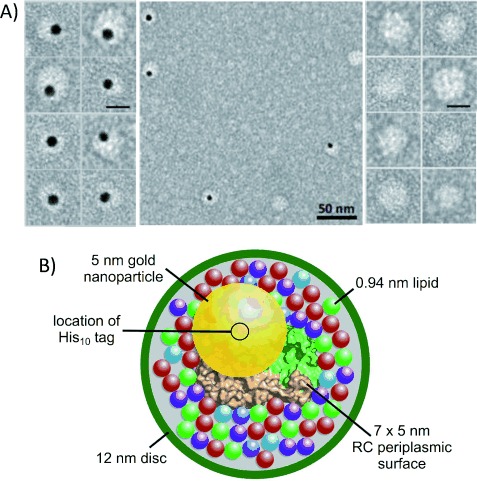
Negative stain TEM and model of SMA-purified RC nanodiscs. A) The galleries show enlarged views (scale bar 10 nm) of individual nanodiscs with (left) and without (right) 5 nm gold nanoparticles attached to the His_10_ tag of the RC. B) Model of a nanodisc viewed orthogonal to the roughly elliptical periplasmic surface of the RC. Distances are diameters of the protein (lime/beige), each of five species of lipid (75 in total—colors and proportions as Figure 3 B), a gold nanoparticle (yellow) and an overall nanodisc (green). The gold nanoparticle is centered on the point of connection of the His_10_ tag to the periplasmic surface of the RC (red, circled).

Thin-layer chromatography (TLC) of extracts of RC native membranes (Figure 3 A) identified phosphatidylethanolamine (PE), phosphatidylcholine (PC), cardiolipin (CL), phosphatidylglycerol (PG), and sulphoquinovosyl diacylglycerol (SQDG) as the principal lipids, in agreement with previous studies on wild-type strains of *Rba. sphaeroides.*^18^ In the SMA/lipid/RC nanodiscs, the same five lipids were found in similar proportions (Figure 3 B), supporting the usefulness of nanodiscs as mimics of the membrane environment for biophysical analysis of a membrane protein in the pure state. Analysis of the phosphate content of SMA-purified RCs produced an estimate of around 150 lipids per RC, corresponding to a nanodisc with an average of three layers of lipids around the RC, assuming that a typical lipid occupies an area of approximately 0.7 nm^2^ 19 (Figure 2 B). No lipids could be detected by TLC in samples of RCs solubilized in either DDM or LDAO at up to a five-fold higher protein concentration, showing that they had been stripped away to below detectable levels (Figure 3 A).

**Figure 3:**
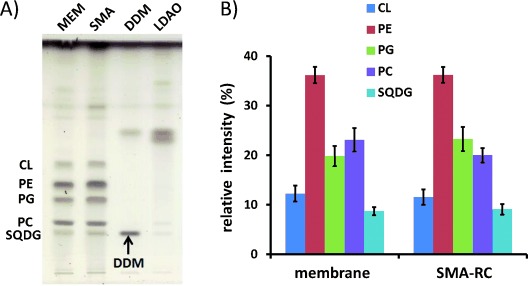
Lipid content of intact membranes and purified RCs. A) Lipid profiles determined by TLC. Lipids were identified by running pure samples of each as a standard (not shown). Bands above the labeled lipids are attributed to RC pigments. DDM was visualized but LDAO did not stain. Additional bands in the DDM and LDAO profiles are unidentified. B) Relative populations of lipids in intact membranes and SMA/lipid nanodiscs from six independent lipid extractions, quantified by densitometry.

Modulation of the functional properties of RCs by the lipid/detergent/copolymer environment was investigated through two readily measurable parameters. First, the mid-point oxidation potential was determined for the bacteriochlorophyll pair (P865) that form the primary donor of electrons during charge separation (see Section 2 of the Supporting Information). This was achieved by monitoring the intensity of their ground-state absorbance band at 865 nm during cycling of the applied potential in an electrochemical cell;^20^ this band bleaches when P865 is oxidized (inset to Figure 4 A). The mid-point potential obtained for RCs in SMA/lipid nanodiscs, 443 mV, was similar to the 449 mV obtained for RCs in native membranes and significantly lower than the 465 and 485 mV obtained for RCs in DDM and LDAO, respectively (Figure 4 A).

**Figure 4:**
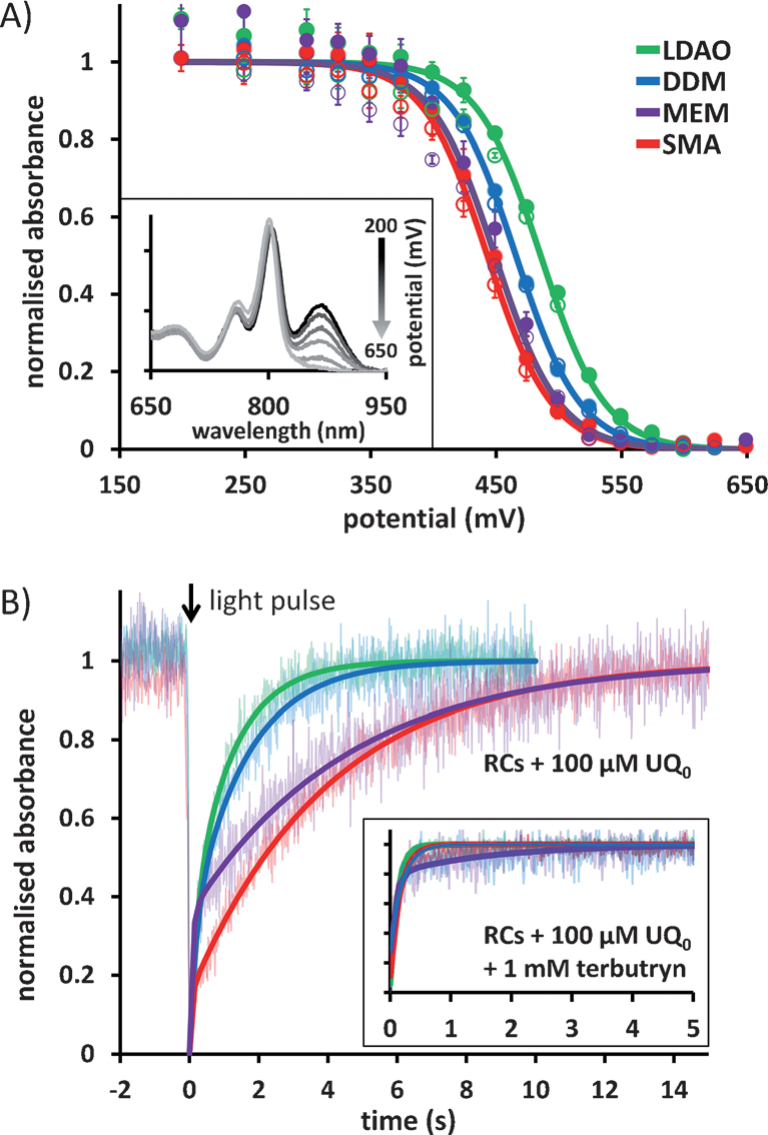
Effect of lipid/detergent/copolymer environment on RC functional properties. A) Average variation of P865 absorbance over three oxidative (filled circles) and reductive (closed circles) titrations of applied potential, with the standard error for each data point. Solid lines show fits with the Nernst equation (*n*=1); mid-point potentials are reported in the text and were associated with a standard error of ±3 mV or less. Inset: Absorbance spectra for SMA-solubilized RCs at progressively increasing applied potentials (black to light gray), showing oxidative bleaching of the band at 865 nm. B) Averaged traces (faded lines) showing photobleaching and recovery of the same absorbance band at 865 nm, normalized to the maximal extent of the initial bleach for comparison. Inset: recovery portion of transients recorded in the presence of 1 mm terbutryn.

Second, a short flash of light was used to form the charge-separated state P865^+^Quinone^−^, and the rate of recombination of this radical pair was monitored through the recovery of bleaching of the same ground-state absorbance band of P865 (Figure 4 B). Photoexcitation triggers membrane-spanning charge separation on a picosecond timescale, the electron arriving first on the tightly bound Q_A_ ubiquinone acceptor and then moving on to, if present, the dissociable Q_B_ ubiquinone acceptor (see the Supporting Information, Section 2 and Figure S2). In LDAO-solubilized RCs, the P865^+^Q_B_^−^ radical pair recombines with a lifetime of around 1 s, whereas, if the Q_B_ quinone is not present, the P865^+^Q_A_^−^ radical pair recombines with a lifetime of around 100 ms.11a, 21 Furthermore, the recombination of P865^+^Q_B_^−^ is slower in RCs in a lipid bilayer than in RCs in detergent by a factor of 1.5 to 3.5, depending on the lipid system used.^15^ The kinetics of P865^+^Quinone^−^ recombination are therefore dependent on the occupancy of the Q_B_ site and on the detergent/lipid environment of the RC.

In the present study, charge recombination was monitored in the presence of a 7.5-fold molar excess of UQ_0_ to largely reconstitute ubiquinone binding at the Q_B_ site. The overall rate of charge recombination in RCs in SMA/lipid nanodiscs was similar to that of RCs in native membranes, and in both cases was clearly slower than in RCs in LDAO or DDM (Figure 4 B). Parameters from biexponential fits to the data in Figure 4 B are shown Table S1 in the Supporting Information; based on the lifetime of the dominant slower component, *τ*_2_, recombination in membranes or SMA/lipid nanodiscs (4.5 and 4.0 s, respectively) was between 2.5 and 4 times slower than in RCs in detergent (1.2 s for LDAO, 1.6 s for DDM). The slower P865^+^Q_B_^−^ charge recombination in RCs in intact membranes is a well-known phenomenon that is attributable to small environmental modulations of redox potential and/or reorganization energy. The fact that this modulation is preserved in RCs in SMA/lipid nanodiscs underlines the way in which they preserve a membrane-like environment for the purified protein.

One application of purple bacterial RCs is their use as the active element in a photoelectrochemical biosensor for herbicides such as terbutryn that block photosynthetic electron transfer in plants by binding to the equivalent Q_B_ site in photosystem II.^22^ To be useful in this way, it is necessary for the intramembrane Q_B_ site in the bacterial RC to be accessible to herbicide molecules. In the present study, the addition of terbutryn led to almost complete loss of the slow phase of recombination from Q_B_^−^, yielding a single component with a time constant of less than 200 ms, attributable to the recombination of P865^+^Q_A_^−^ (inset to Figure 4 B and Table S1 in the Supporting Information). This result shows that accessibility of the Q_B_ site to inhibitors is not occluded by housing RCs in SMA/lipid nanodiscs.

Successful exploitation of *Rba. sphaeroides* RCs in applications requires the protein to be stable under illumination. The integrity of the protein can be monitored through the bacteriochlorin absorbance bands at 803 and 760 nm, which show distinctive decreases in amplitude as the protein unfolds (see inset to Figure 5 A; note that the band at 865 nm photobleaches immediately on strong illumination). As in previous work,^23^ degradation of the protein in response to light stress was assayed by simply monitoring the decrease in absorbance at 803 nm during incubation of RCs at room temperature in the light. The rate of photodegradation of RCs in SMA/lipid nanodiscs was similar to that for RCs in DDM, which has a reputation for being a “stabilizing” detergent, but was markedly slower than for either RCs in native membranes or LDAO micelles (Figure 5 A). Although LDAO is used extensively for work on RCs, having the advantage that it is relatively inexpensive, it is not particularly stabilizing, and so the difference in the rate of photodegradation of RCs in DDM and LDAO was not a surprise.

**Figure 5:**
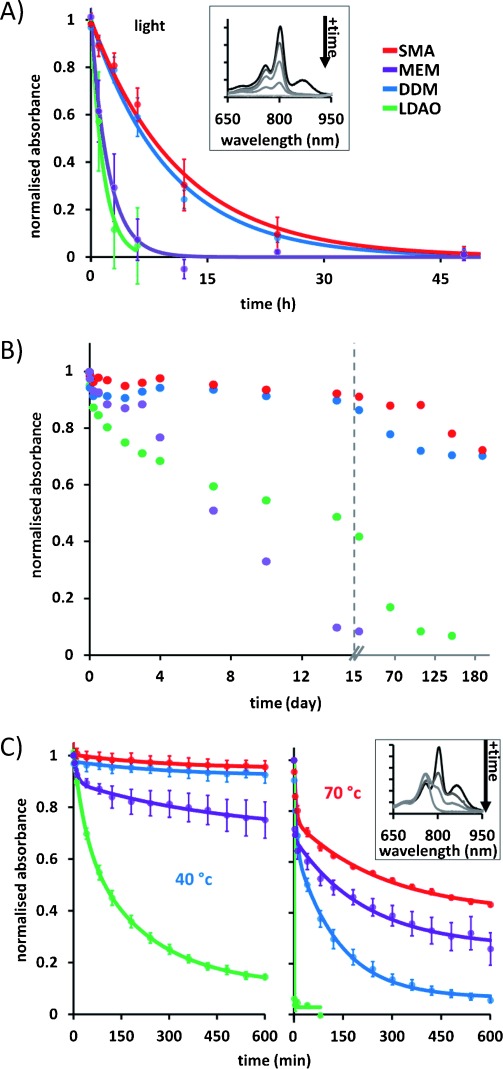
Stability of RCs under stress monitored through absorbance decreases at 803 nm. A) Photostability of RCs at room temperature in the light. Circles show averages from three time courses with standard errors. Solid lines show fits to a single exponential decay as a guide to the eye. Inset: absorbance spectra of SMA solubilized RCs before (black) and after (gray) strong illumination for 6, 12, 24, and 48 h. B) Data for duplicate RC preparations at room temperature in the dark. Color coding as for panel A. C) Thermal stability during a time course at 40 °C (left) or 70 °C (right). Circles show average data from three time courses with standard errors. Solid lines show fits to a double exponential decay. Inset: absorbance spectra of SMA-solubilized RCs before (black) and after (gray) heating at 80 °C for 2.5, 10, and 300 min.

More of a surprise was the relative instability of RCs embedded in native membranes when exposed to light stress. This instability was also observed for control samples incubated at room temperature in the dark for a period of weeks (Figure 5 B). Over the first three days, the stability of RCs in native membranes was similar to that of RCs in nanodiscs or in DDM, but then steadily declined over the next two weeks. A possible explanation for the instability of the RCs in membranes is that protein damage is induced by the membrane environment through autocatalytic oxidation of the lipids. Although such lipid oxidation would also take place in the nanodiscs, it would be slowed down because the lipid material is divided over many membrane nanoparticles, which isolate the approximately 150 lipids occupying each nanodisc, reducing any autocatalytic propagation. Also other redox proteins present in native membranes that may play a role in the generation of the reactive oxygen species that initiate damage will not be present in the RC nanodiscs.

This finding highlights an additional aspect of the protection offered by the nanodisc environment to the encased protein. Intact photosynthetic membranes have been considered as a more stable alternative to detergent-purified proteins for the generation of photocurrents.^24^ Our results indicate that the use of nanodiscs may provide the best of both worlds by offering a protective membrane-like environment that is less prone to membrane damage. Under long-term storage at room temperature (20–25 °C) in the dark, membrane-embedded RCs showed complete degradation after 21 days and LDAO RCs after 100 days (Figure 5 B). However, RCs in nanodiscs or DDM had retained ≈70 % of their native absorbance after six months.

Another key issue is stability under thermal stress, which can also be assessed using the RC absorbance spectrum. During heating, the bands at 865 and 803 nm drop in intensity as the protein unfolds, while a band attributable to released bacteriochlorophyll will appear at around 760 nm (see inset to Figure 5 C and previous accounts^23^). Assays showed that the stability of RCs in SMA/lipid nanodiscs is comparable to that of RCs in intact membranes or in DDM micelles at temperatures between 40 and 70 °C (Figure 5 C), but that RCs in LDAO micelles are markedly less stable. The RCs in nanodiscs lost their native absorbance more slowly than RCs in DDM or intact membranes at all temperatures, the effect getting stronger as the temperature was increased. Again this highlights the strong protective environment that the SMA/lipid nanodiscs offer to the RC.

To summarize, the data outlined above show that it is possible to use a SMA copolymer to solubilize a His-tagged photovoltaic integral protein from a native membrane while maintaining its immediate native lipid environment, and purify it using standard affinity and size-exclusion chromatography in the complete absence of detergent. The SMA/lipid nanodiscs offer a membrane-like environment to RCs that preserves native functional properties. Notably, the RC protein is generally more stable when encased in the nanodiscs than in detergent solution or even in the native membrane. This is of particular interest, given that a major barrier to the exploitation of membrane proteins for technological applications is their limited stability under conditions of stress. The characteristics of currents generated by RC/SMA/lipid nanodiscs interfaced in a variety of ways with electrode materials are currently being explored, with focus on their stability and longevity.

Finally, it is important to emphasize the key roles that lipids play in membrane protein function and stability. A significant advantage of the SMA approach to membrane protein purification is that it enables the micromembrane environment to be maintained and analyzed without interference from detergent. Furthermore, given the preservation of native function evidenced above, it is possible that purification of membrane proteins using SMA could become the standard tool for biophysical studies of membrane proteins, and that the copolymer will greatly facilitate the wider use of these lipid/protein nanodiscs in biohybrid devices.
