# Supplementary material for: Bacterial Reaction Centers Purified with Styrene Maleic Acid Copolymer Retain Native Membrane Functional Properties and Display Enhanced Stability
Source: Angew Chem Int Ed Engl. 2014 Sep 11;53(44):11803–7. doi: 10.1002/anie.201406412 (PMC4271668; doi:10.1002/anie.201406412)
Supplement: Supplementary file 1 [file anie0053-11803-sd1.pdf]

Angewandte Chemie
Eine Zeitschrift der Gesellschaft Deutscher Chemiker

Supporting Information

© Wiley-VCH 2014

69451 Weinheim, Germany

# Bacterial Reaction Centers Purified with Styrene Maleic Acid Copolymer Retain Native Membrane Functional Properties and Display Enhanced Stability**

*David J. K. Swainsbury, Stefan Scheidelaar, Rienk van Grondelle, J. Antoinette Killian,* and Michael R. Jones**

anie_201406412_sm_miscellaneous_information.pdf

# Table of Contents

**1. Experimental Procedures**

1.1. Preparation of styrene maleic acid

1.2. Purification of RCs in nanodiscs

1.3. Purification of LDAO and DDM RCs

1.4. Purity of RC preparations (Figure S1. Absorbance spectra of pure RCs)

1.5. Preparation of native membranes for spectroscopy/potentiometry

1.6. RC ground state absorbance spectra

1.7. Dynamic light scattering

1.8. Transmission electron microscopy

1.9. Thin layer chromatography and lipid analysis

1.10. Redox potentiometry

1.11. Measurement of $P^+Q_B^-$ recombination kinetics

1.12. Thermal stability of RCs

1.13. Photostability of RCs

**2. Reaction Center Charge Recombination**

Figure S2. Charge recombination.

Table S1. Effect of RC environment on the rate of photoinduced charge recombination.

# 1. Experimental Procedures

**1.1. Preparation of styrene maleic acid.** Styrene maleic anhydride (SMA 2000, MW 7500, polydispersity index 2.5, ratio of 2:1 styrene to maleic anhydride) was a kind gift of Cray Valley, USA. SMA 2000 was refluxed for 6 hours at 5 % (w/v) in 1 M KOH, and the resulting hydrolyzed polymer was precipitated by addition of 6 M HCl to a final concentration of 1.1 M HCl. The precipitate was centrifuged at 16,000 x g for 30 minutes, the supernatant was removed and the polymer was washed by resuspension in an equal volume of 100 mM HCl. This cycle of centrifugation and resuspension was repeated five times to remove any traces of salt, and the styrene maleic acid (SMA) copolymer was then freeze dried. Completion of the reaction was confirmed by FTIR spectroscopy by the disappearance of the maleic anhydride carboxyl signal at 1780 $cm^{-1}$ and the appearance of the maleic acid carbonyl signal at 1570 $cm^{-1}$. A solution of 6 % (w/v) SMA in 50 mM Tris (pH 8.0) was used for RC purification.

**1.2. Purification of RCs in nanodiscs.** Cells of *Rba. sphaeroides* lacking light harvesting proteins and expressing RCs with a $His_{10}$ tag[S1] were grown under dark/semiaerobic conditions[S2]. Cells harvested from 9 L of culture medium by centrifugation (5,000 x g for 20 mins) were suspended in 60 mL of 20 mM Tris (pH 8.0) containing a few crystals of DNAse I and two cOmplete EDTA-free protease inhibitor tablets (Roche). Cells were lysed in a Constant Systems cell disruptor at 20,000 psi, cell debris being removed by centrifugation at 26,890 x g for 15 min at 4 °C. Aliquots of 6 % (w/v) SMA in 50 mM Tris (pH 8.0) and of 5 M NaCl were added to the membranes in the supernatant to give final concentrations of 1.5 % and 100 mM, respectively, in a total volume of 250 mL. This solution was incubated at room temperature in the dark for 1 h with gentle stirring, and membrane debris was then removed by ultracentrifugation at 100,000 x g at 4 °C for 30 minutes. The efficiency of RC solubilization by 6 % SMA was comparable to that achieved using a standard detergent-based protocol (0.5 % LDAO - see below). A 20 mL HisPrep FF 16/60 chromatography column (GE Healthcare) was equilibrated with 20 mM Tris/200 mM NaCl/10 mM imidazole at pH 8 (equilibration buffer) and the SMA-solubilized RCs were loaded by cycling the supernatant through the column overnight at a flow rate of approximately 3 mL $min^{-1}$. After loading, the column was washed with 20 column volumes of equilibration buffer, and the protein eluted by the addition of 20 mM Tris /200 mM NaCl/500 mM imidazole at pH 8. Fractions containing RCs were pooled and the SMA-solubilized RCs were further purified by passage through a Superdex 200 16/600 gel filtration column (GE Healthcare) pre-equilibrated with 20 mM Tris (pH 8). RC fractions with an absorbance ratio at 280 nm and 803 nm of ~1.5 (see below) were pooled, concentrated and stored at -20 °C until required.

**1.3. Purification of LDAO and DDM RCs.** LDAO RCs were prepared in essentially the same way as SMA solubilized RCs except that extraction of the RCs from membranes was achieved with final concentrations of 0.5 % LDAO and 200 mM NaCl, and 0.1 % LDAO was included in all buffers used for purification. DDM RCs were prepared by the same method, except that the final gel filtration step was performed in 20 mM Tris (pH 8) containing 0.04% DDM to exchange LDAO for DDM. For all subsequent measurements the buffers used to dilute LDAO or DDM RCs were supplemented with 0.1% LDAO or 0.04% DDM, respectively.

**1.4. Purity of RC preparations.** Purity of RCs was assessed by UV/vis absorbance spectroscopy as described by Okamura and co-workers[15]. Column fractions containing LDAO or DDM purified RCs with a ratio of protein absorbance at 280 nm to bacteriochlorophyll absorbance at 802 nm of ~1.3 were retained for use (Figure S1, green and blue). Purity of these RC preparations was confirmed by SDS-PAGE, the only bands detected being the three constituent polypeptides of the RC (data not shown). The same spectroscopic assay was used during purification of SMA-solubilised RCs, and it was found that at the same degree of purity as assessed by SDS-PAGE these RCs had a $A_{280}/A_{802}$ ratio of ~1.5, and a lower value could not be obtained (Figure S1, red). The source of the additional absorbance around 280 nm was not determined but could be attributable to the SMA copolymer, ubiquinone or carotenoid.

The RC has a single spheroidenone carotenoid with a broad absorbance band between 400 and 550 nm. Compared to detergent-solubilized RCs, some fractions of pure RCs in SMA/lipid nanodiscs showed additional carotenoid absorbance in this region (Figure S1, red compared with blue/green); this is consistent with the presence of free carotenoid in photosynthetic membranes from antenna-deficient strains[12]. Such carotenoid is washed away during detergent purification, but in some fractions a small amount appeared to have been retained in the protein/lipid nanodisc during SMA purification. The lower 865 nm band in the absorbance spectrum of SMA-solubilized RCs in Figure S1 is attributable to partial oxidation of the P865 bacteriochlorophylls under ambient conditions during purification. The fact that this was more pronounced for SMA-solubilized RCs than detergent-solubilized RCs is consistent with the lower P865/P865$^+$ redox potential in the former (see main text). This partial bleaching was not present in the spectra reported in Figure 1B in the main text due the presence of sodium ascorbate which reduces any P865$^+$.

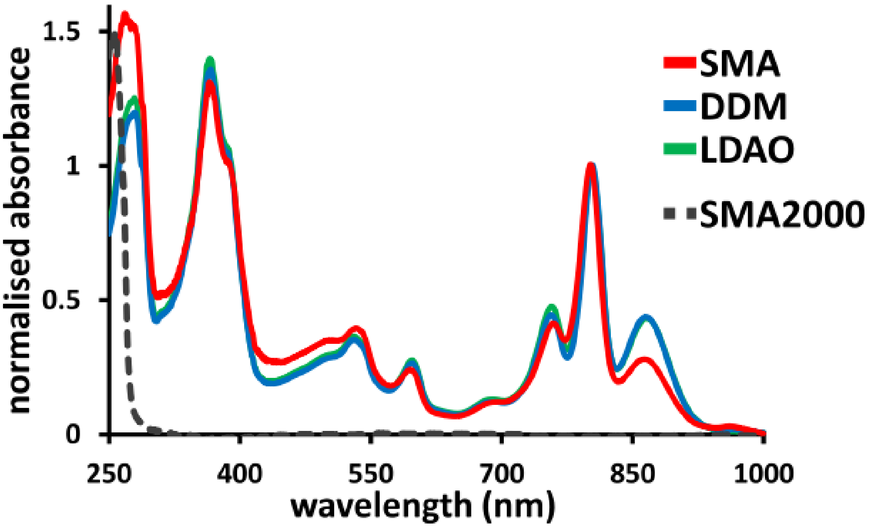


**Figure S1. Absorbance spectra of pure RCs.** Spectra are for column fractions from gel filtration chromatography, recorded without addition of sodium ascorbate.

**1.5. Preparation of native membranes for spectroscopy/potentiometry.** A 1.5 L volume of cells of the antenna-deficient strain of *Rba. sphaeroides* expressing $His_{10}$-tagged RCs was grown as described above and harvested by centrifugation. Pelleted cells were suspended in 20 mM Tris (pH 8) containing a few crystals of DNAse I and lysed by passing twice through a French pressure cell at 20,000 psi. Cell debris was removed by centrifugation at 26,890 x g for 15 min at 4 °C, the lysate overlaid on a cushion of 60 % w/v sucrose, and centrifuged at 100,000 x g for 2 h. The tight membrane band on top of the sucrose layer was collected, the sucrose removed by dialysis against 20 mM Tris (pH 8), and the membrane suspension stored at -20 °C until required.

**1.6. RC ground state absorbance spectra.** RC concentrations were calculated using an extinction coefficient at 802 nm of $2.88 \times 10^5$ $M^{-1}$ $cm^{-1}$[S3]. RCs were diluted to 0.5 µM in 20 mM Tris (pH 8) (containing detergent if appropriate). Sodium ascorbate (2 mM) was also present to prevent oxidative bleaching of the RC absorbance band at 865 nm. Absorbance spectra were recorded between 300 and 1000 nm after dark adaptation of each sample for 30 mins. The spectrum of membrane-embedded RCs was distorted by background light scatter, and this was corrected for by subtraction of a scatter curve comprising a modified Rayleigh function where Absorbance = $\lambda^{-2.6}$. This curve was calculated assuming that the absorbance at 950 nm is zero. The points at 950 and 650 nm were used to calculate the scatter curve and an offset of 0.07 was applied at 650 nm to match the values for the other conditions. The scattering power was reduced from -4 to -2.6 to give a better match in the 600-700 nm range, which is not expected to be altered by the RC environment. For comparison, spectra were then normalised to the strongest absorbance band in the near-infrared at ~802 nm.

**1.7. Dynamic light scattering.** RCs were diluted to 2 µM in 20 mM Tris (pH 8)/200 mM NaCl. DLS measurements were carried out in a 200 µL micro cuvette (Hellma) in a Zetasizer Nano ZS (Malvern Instruments). Correlation curves were produced from three sets of twelve spectra and processed using the Zetasizer software package. Particle diameters reported in the text were associated with a percentage mass of ≥ 99.9 %.

**1.8. Transmission electron microscopy.** A 5 µL aliquot of SMA solubilized RCs (8 mg $mL^{-1}$) was adsorbed onto a glow discharged copper grid for 2 minutes. Excess sample was removed with filter paper, and 10 µL of a 0.5 µM solution of 5 nm Ni-NTA functionalized gold nanoparticles were adsorbed onto the SMA solubilized RC copper grid for 20 minutes. Excess liquid was removed with filter paper and the copper grid was washed twice with water for 15 s. The copper grid was then stained with 2 % ammonium molybdate for 45 s and excess liquid was removed with filter paper. The copper grid was air-dried and examined using a Tecnai 12 Philips electron microscope operating at an acceleration voltage of 120 kV.

**1.9. Thin layer chromatography and lipid analysis.** Six independent lipid extractions were performed from the same batch of purified RCs or membranes. Lipids were extracted from native membranes, SMA solubilized RCs, RCs in DDM micelles and RCs in LDAO micelles by the Bligh and Dyer method[S4]. Extracted lipids were deposited onto a silica TLC plate (MACHEREY NAGEL GmbH & co) using a Linomat 5 sample applicator

(Camag). The TLC plate was developed using a solution of chloroform:methanol:acetic acid:water (85:15:10:3.5) in an ADC2 Automatic Development Chamber (Camag). Lipids were visualized by dipping the plate into a methanol solution of 10 % copper(II) sulfate in 8 % sulfuric acid (98 %), and 8 % phosphoric acid (85 %) and then drying the plate by heating at 130˚C for 12 min. Copper(II) charring was used because it yields excellent signal-to-noise ratio in intensity, much better than for instance sulfuric acid charring[S5]. Only LDAO could not be visualized by copper(II) charring. Relative intensities were determined by densitometry using Quantity One (BioRad). The image shown in Figure 3A is slightly contrast enhanced (Adobe Photoshop CS6).

Lipid phosphate concentration was measured by the Rouser method[S6] using samples of known RC concentration. Amounts of lipid per nanodisc assumed that every His-tag purified nanodisc contains a single RC. It should be noted that the SQDG lipid content does not contribute because it does not contain a phosphate. On the other hand, cardiolipin (CL) adds two phosphates per molecule. The amounts of these two lipids in the SMA nanodiscs were similar, and so these effects approximately cancel out.

**1.10. Redox potentiometry.** Determination of the mid-point redox potential of the RC primary electron donor P865 was based on the method by Moss *et al.*[18]. RCs were diluted to 20 µM in 50 mM $KH_2PO_4$ (pH 7.5)/1 M KCl/10 mM $UQ_0$/10 mM potassium ferricyanide/2 mM TMPD (*N*,*N*,*N*',*N*'-tetramethyl-p-phenylenediamine)/20% v/v glycerol, with detergent if appropriate. An aliquot was placed in a sealed 200 µL transparent cell with a 0.3 mm path length fitted with a platinum gauze working electrode, platinum counter electrode and Ag/AgCl reference electrode. A series of potentials between 0 and 450 mV versus the reference electrode were applied and at each potential absorbance spectra were collected continuously between 650 and 1000 nm until there was no change in lineshape, the final spectrum at each potential being used for the subsequent analysis. In each titration the mid-point potential was determined by fitting the absorbance at 865 nm as a function of applied potential to a one electron Nernst function in Origin 8 (OriginLab). The standard error reported by this fitting process was typically no greater than ± 3 mV. The instrument was calibrated by carrying out a titration in the absence of RC protein, monitoring the absorbance band at 410 nm arising from ferricyanide and using the known mid-point potential of 430 mV for the ferricyanide/ferrocyanide couple.

**1.11. Measurement of $P^+Q_B^-$ recombination kinetics.** Measurements of P865 photo-oxidation and re-reduction through recombination were performed using a Cary60 spectrophotometer connected to an external CUV 1 cm cuvette holder (Ocean Optics) via a pair of optical fibres. Light pulses of 50 ms duration were applied to the sample at 90° to the pulsed measuring beam using an HL-2000-FHSA shutter controlled white light source (Ocean Optics) delivering approximately 25 W $m^{-2}$ light intensity at the cuvette surface via an optical fiber; rapid shutter opening and closing was triggered by a TGP110 pulse generator (tti instruments). Solutions of 13.2 µM RCs were prepared in 20 mM Tris (pH 8) containing 100 µM $UQ_0$ and detergent if appropriate. RC samples were loaded in to a 3 x 3 mm fluorescence cuvette (Hellma) and the absorbance at 865 nm was measured before and for 20 s after delivery of the excitation pulse. Eight transients were recorded for each sample and averaged before analysis. Charge recombination kinetics were fitted with a single or double exponential function in Origin 8 (OriginLab). Parameters from the kinetic fits are shown in Table S1, below.

**1.12. Thermal stability of RCs.** Assays of thermal stability were performed in a Cary60 spectrophotometer equipped with a temperature-controlled multi-cell holder. Semi-micro cuvettes were filled with 1.6 ml of 20 mM Tris (pH 8) (and detergent if appropriate) that had been pre-heated to the desired temperature. Melts were initiated by rapidly mixing in adding 150 µL of RC solution in the same buffer to give a final RC concentration of 0.5 µM, and sealing the cuvette with a stopper, excluding any air bubbles. Absorbance spectra between 600 and 1000 nm were recorded at intervals after mixing from 1 min to 10 h.

**1.13. Photostability of RCs.** RCs prepared with LDAO, DDM, SMA or in native membranes were diluted to 0.5 µM in 20 mM Tris (pH 8) (with detergent if appropriate) and used to completely fill a pair of stoppered cuvettes. Initial absorbance spectra were recorded and one set of cuvettes was placed in front of a 100 W white light source shielded by a water filter, which delivered a light intensity of approximately 90 mW $cm^{-2}$ at each cuvette surface. The water filter prevented heating of the cuvettes above ambient. The second set of cuvettes was placed in the dark as a control. Absorbance spectra were recorded between 600 and 1000 nm at intervals.

## 2. Reaction Center Charge Recombination

As shown in Figure S2, the initial steps in the mechanism of the *Rba. sphaeroides* RC involve photochemical charge separation (blue arrow) to create the radical pair $P865^{+}Q_A^{-}$ on a picosecond time scale. If the $Q_B$ binding site is not occupied by an oxidized ubiquinone (panel on left) $P865^{+}Q_A^{-}$ recombines with a lifetime of around 100 ms. If the $Q_B$ site is occupied (panel on right) the electron is transferred from $Q_A^{-}$ to $Q_B$ on a microsecond time scale, creating the radical pair $P865^{+}Q_B^{-}$. In the absence of additional external electron donors or acceptors, or further photoexcitation, $P865^{+}Q_B^{-}$ recombines with a lifetime that varies between 1 and 5 s, depending on the detergent/lipid environment of the RC. This process can be monitored through the absorbance band attributable to the ground state of the P865 bacteriochlorophyll pair; this band is bleached when P865 is oxidized and recovers as the electron returns from $Q_A^{-}$ or $Q_B^{-}$.

In the present study, charge recombination was compared in RCs in native membranes, SMA/lipid nanodiscs and LDAO or DDM micelles. Data from the kinetic fits in Figure 4B in the main text are shown in Table S1, below. The fast phase ($\tau_1$, $A_1$) represents recombination of $P865^{+}Q_A^{-}$ in RCs where $Q_B$ is either absent or the site is blocked by the inhibitor terbutryn. The slow phase ($\tau_2$, $A_2$) represents recombination of $P865^{+}Q_B^{-}$.

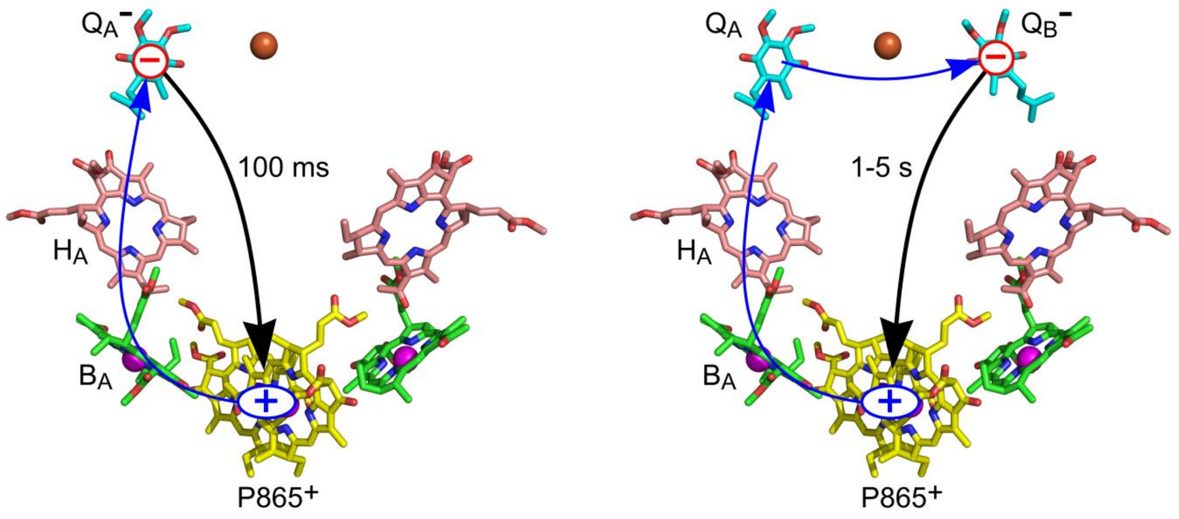


**Figure S2. Charge recombination.** Recombination of $P865^{+}Q_A^{-}$ or $P865^{+}Q_B^{-}$ (black arrows) follows light-induced charge separation (blue arrows). The panels show the P865 bacteriochlorophyll pair (yellow carbons), two accessory bacteriochlorophylls (green carbons), two bacteriopheophytins (pink carbons) and either one or two ubiquinones (cyan carbons). Other atoms are oxygen (red), nitrogen (blue), magnesium (magenta spheres) and iron (brown sphere). Charge separation takes place along only one of the two cofactor branches, via a bacteriochlorophyll ($B_A$) and bacteriopheophytin ($H_A$). Hydrocarbon side chains of the RC cofactors and the carotenoid have been omitted for clarity.

**Table S1. Effect of RC environment on the lifetimes (τ) and amplitudes (A) of components of photoinduced charge recombination.**

| RC | $\tau_1$[a] s | $A_1$[a] % | $\tau_2$ s | $A_2$ % |
|---|---|---|---|---|
| LDAO | 0.20 ± 0.03 | 33 ± 3 | 1.19 ± 0.04 | 67 ± 3 |
| DDM | 0.18 ± 0.03 | 31 ± 3 | 1.58 ± 0.06 | 69 ± 2 |
| nanodisc | 0.06 ± 0.02 | 15 ± 3 | 4.03 ± 0.06 | 85.1 ± 0.4 |
| membranes | 0.07 ± 0.01 | 35 ± 4 | 4.52 ± 0.15 | 65 ± 1 |
| LDAO + terb[b] | 0.117 ± 0.003 | 100 ± 2 | | |
| DDM + terb[b] | 0.195 ± 0.016 | 100 ± 5 | | |
| nanodisc + terb[b] | 0.168 ± 0.005 | 100 ± 2 | | |
| membranes + terb[b] | 0.085 ± 0.017 | 72 ± 8 | 1.64 ± 0.23 | 28 ± 3 |

[a] The significance of the precise lifetime and amplitude of the fast phase ($\tau_1$, $A_1$) should be treated with caution as the ~50 ms excitation pulse is long relative to this component and noise levels in the averaged kinetic traces are high, especially for RCs in intact membranes.

[b] 1mM terbutryn added to inhibit electron transfer to $Q_B$

## Supporting References


[S1] D. J. K. Swainsbury, V. M. Friebe, R. N. Frese, M. R. Jones, *Biosens. Bioelectron.* **2014**, *58*, 172-178.

[S2] M. R. Jones, M. Heer-Dawson, T. A. Mattioli, C. N. Hunter, B. Robert, *FEBS Letters* **1994**, *339*, 18-24.

[S3] S. C. Straley, W. W. Parson, D. C. Mauzerall, R. K. Clayton, *Biochim. Biophys. Acta* **1973**, *305*, 597-609.

[S4] E. G. Bligh, W. J. Dyer, *Can. J. Biochem. Physiol.* **1959**, *37*, 911-917.

[S5] A. A. Entezami, B. J. Venables, K. E. Daugherty, *J. Chromatogr.* **1987**, *387*, 323-331.

[S6] G. Rouser, S. Fleischer, A. Yamamoto, *Lipid* **1970**, *5*, 494-496.

[S7] K. Gibasiewicz, M. Pajzderska, J. A. Potter, P. K. Fyfe, A. Dobek, K. Brettel, M. R. Jones, *J. Phys. Chem. B* **2011**, *115*, 13037-13050.
